# Qubit lattice coherence induced by electromagnetic pulses in superconducting metamaterials

**DOI:** 10.1038/srep29374

**Published:** 2016-07-12

**Authors:** Z. Ivić, N. Lazarides, G. P. Tsironis

**Affiliations:** 1 Crete Center for Quantum Complexity and Nanotechnology, Department of Physics, University of Crete, P. O. Box 2208, 71003 Heraklion, Greece; 2 University of Belgrade, “Vinča” Institute of Nuclear Sciences, Laboratory for Theoretical and Condensed Matter Physics, P.O. Box 522, 11001 Belgrade, Serbia; 3 National University of Science and Technology MISiS, Leninsky prosp. 4, Moscow, 119049, Russia; 4 Institute of Electronic Structure and Laser, Foundation for Research and Technology–Hellas, P.O. Box 1527, 71110 Heraklion, Greece; 5 Department of Physics, School of Science and Technology, Nazarbayev University, 53 Kabanbay Batyr Ave., Astana 010000, Kazakhstan

## Abstract

Quantum bits (qubits) are at the heart of quantum information processing schemes. Currently, solid-state qubits, and in particular the superconducting ones, seem to satisfy the requirements for being the building blocks of viable quantum computers, since they exhibit relatively long coherence times, extremely low dissipation, and scalability. The possibility of achieving quantum coherence in macroscopic circuits comprising Josephson junctions, envisioned by Legett in the 1980’s, was demonstrated for the first time in a charge qubit; since then, the exploitation of macroscopic quantum effects in low-capacitance Josephson junction circuits allowed for the realization of several kinds of superconducting qubits. Furthermore, coupling between qubits has been successfully achieved that was followed by the construction of multiple-qubit logic gates and the implementation of several algorithms. Here it is demonstrated that induced qubit lattice coherence as well as two remarkable quantum coherent optical phenomena, i.e., self-induced transparency and Dicke-type superradiance, may occur during light-pulse propagation in quantum metamaterials comprising superconducting charge qubits. The generated qubit lattice pulse forms a compound ”quantum breather” that propagates in synchrony with the electromagnetic pulse. The experimental confirmation of such effects in superconducting quantum metamaterials may open a new pathway to potentially powerful quantum computing.

Quantum simulation, that holds promises of solving particular problems exponentially faster than any classical computer, is a rapidly expanding field of research^1^,^2^,^3^. The information in quantum computers is stored in quantum bits or qubits, which have found several physical realizations; quantum simulators have been nowadays realized and/or proposed that employ trapped ions^4^, ultracold quantum gases^5^, photonic systems^6^, quantum dots^7^, and superconducting circuits^1^,^8^,^9^. Solid state devices, and in particular those relying on the Josephson effect^10^, are gaining ground as preferable elementary units (qubits) of quantum simulators since they exhibit relatively long coherence times and extremely low dissipation^11^. Several variants of Josephson qubits that utilize either charge or flux or phase degrees of freedom have been proposed for implementing a working quantum computer; the recently anounced, commercially available quantum computer with more than 1000 superconducting qubit CPU, known as D-Wave 2X^*TM*^ (the upgrade of D-Wave Two^*TM*^ with 512 qubits CPU), is clearly a major advancement in this direction. A single superconducting charge qubit (SCQ)^12^ at milikelvin temperatures behaves effectively as an artificial two-level “atom” in which two states, the ground and the first excited ones, are coherently superposed by Josephson coupling. When coupled to an electromagnetic (EM) vector potential, a single SCQ does behave, with respect to the scattering of EM waves, as an atom in space. Indeed, a “single-atom laser” has been realized with an SCQ coupled to a transmission line resonator (“cavity”)^13^. Thus, it would be anticipated that a periodic structure of SCQs demonstrates the properties of a transparent material, at least in a particular frequency band. The idea of building materials comprising artificial “atoms” with engineered properties, i.e., *metamaterials*, and in particular superconducting ones^14^, is currently under active development. *Superconducting quantum metamaterials* (SCQMMs) comprising a large number of qubits could hopefully maintain quantum coherence for times long enough to reveal new, exotic collective properties. The first SCQMM that was only recently implemented comprises 20 flux qubits arranged in a double chain geometry^15^. Furthermore, lasing in the microwave range has been demonstrated theoretically to be triggered in an SCQMM initialized in an easily reachable factorized state^16^.

## Results

### Superconducting Quantum Metamaterial Model

**Figure 1:**
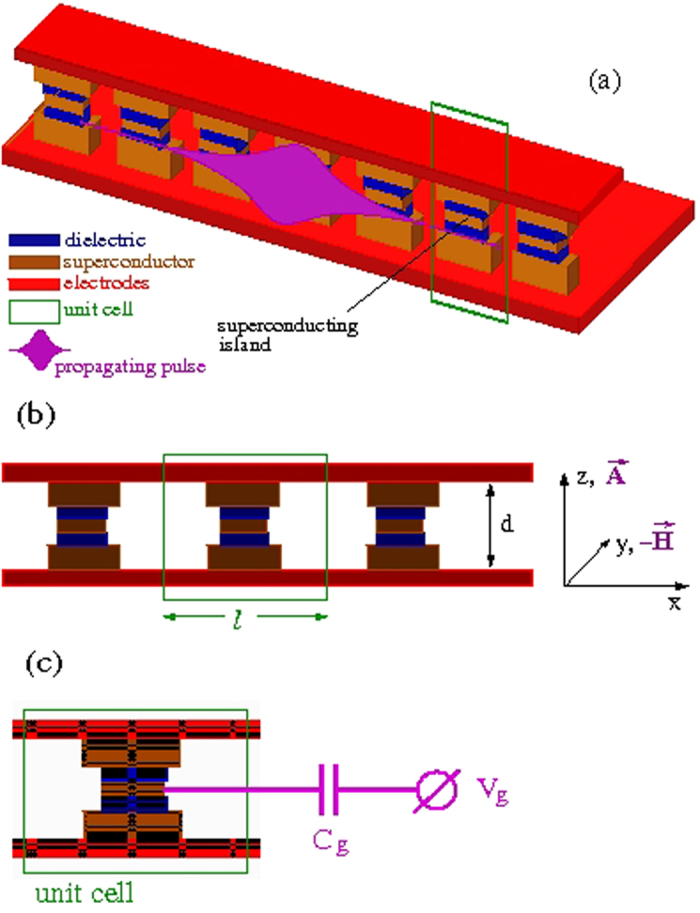
Schematic drawing of a charge qubit superconducting quantum metamaterial (SCQMM). (**a**) The SCQMM comprising an infinite chain of identical charge qubits in a superconducting transmission line. Each qubit consists of a superconducting island that is connected to the electrodes of the transmission line through two Josephson junctions, formed in the regions of the dielectric layers (blue). The propagating electromagnetic vector potential pulse is also shown schematically out of scale. (**b**) The side view of the SCQMM in which the relevant geometrical parameters and the field orientations are indicated. (**c**) A unit cell of the superconducting quantum metamaterial which also shows the control circuitry of the charge qubit, consisting of a gate potential *V*_*g*_ applied to it through the gate capacitor *C*_*g*_.

Consider an infinite, one-dimensional (1D) periodic SCQ array placed in a transmission line (TL) consisting of two superconducting strips of infinite length^17^,^18^ (Fig. 1a,b); each SCQ, in the form of a tiny superconducting island, is connected to each bank of the TL by a Josephson junction (JJ). The control circuitry for each individual SCQ (Fig. 1c), consisting of a gate voltage source *V*_*g*_ coupled to it through a gate capacitor *C*_*g*_, allows for local control of the SCQMM by altering independently the state of each SCQ^19^. The SCQs exploit the nonlinearity of the Josephson effect and the large charging energy resulting from nanofabrication to create artificial mesoscopic two-level systems. A propagating EM field in the superconducting TL gives rise to nontrivial interactions between the SCQs, that are mediated by its photons^20^. Those interactions are of fundamental importance in quantum optics, quantum simulations, and quantum information processing, as well. In what follows, it is demonstrated theoretically that self-induced transparency^21^ and Dicke-type superradiance (collective spontaneous emission)^22^ occur for weak EM fields in that SCQMM structure; the occurence of the former or the latter effect solely depends on the initial state of the SCQ subsystem. Most importantly, self-induced transparent (SIT) or superradiant (SRD) pulses induce quantum coherence effects in the qubit subsystem. In superradiance (resp. self-induced transparency), the initial conditions correspond to a state where the SCQs are all in their excited (resp. ground) state; an extended system exhibiting SRD or SIT effects is often called a coherent amlpifier or attenuator, respectively. These fundamental quantum coherent prosesses have been investigated extensively in connection to one- and two-photon resonant two-level systems. Superradiant effects have been actually observed recently in two-level systems formed by quantum dot arrays^23^ and spin-orbit coupled Bose-Einstein condensates^24^; the latter system features the coupling between momentum states and the collective atomic spin which is analogous to that between the EM field and the atomic spin in the original Dicke model. These results suggest that quantum dots and the atoms in the Bose-Einstein condensate can radiatively interact over long distances. The experimental confirmation of SIT and SRD in extended SCQMM structures may open a new pathway to potentially powerful quantum computing. As a consequence of these effects, the value of the speed of either an SIT or SRD propagating pulse in a SCQMM structure can in principle be engineered through the SCQ parameters^25^, which *is not possible in ordinary resonant media*. From a technological viewpoint, an EM (light) pulse can be regarded as a “bit” of optical information; its slowing down, or even its complete halting for a certain time interval, may be used for data storage in a quantum computer.

In the following, the essential building blocks of the SCQMM model are summarized in a self-contained manner, yet omitting unnecessary calculational details which are presented in the Supplementary Information. The energy per unit cell of the SCQMM structure lying along the *x*–direction, when coupled to an EM vector potential 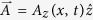, can be readily written as^17^,^18^


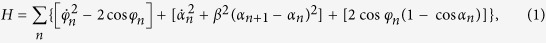


in units of the Josephson energy *E*_*J*_ = Φ_0_*I*_*c*_/(2*πC*), with Φ_0_, *I*_*c*_ and *C* being the magnetic flux quantum, the critical current of the JJ, and the capacitance of the JJ, respectively. In equation (1), *φ*_*n*_ is the superconducting phase on the *n*th island, *β* = (8*πdE*_*J*_)^−1/2^(Φ_0_/2*π*), with *d* being the separation between the electrodes of the superconducting TL, and the overdots denote differentiation with respect to the temporal variable *t*. Assuming EM fields with wavelengths 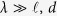, with 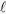 being the distance between neighboring qubits, the EM potential is approximately constant within a unit cell, so that in the centre of the *n*th unit cell 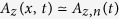. In terms of the discretized EM potential *A*_*z*,*n*_(*t*), the normalized gauge term is *a*_*n*_ = 2*πdA*_*x*,*n*_/Φ_0_. The classical energy expression equation (1) provides a minimal modelling approach for the system under consideration; the three angular brackets in that equation correspond to the energies of the SCQ subsystem, the EM field inside the TL electrodes, and their interaction, respectively. The latter results from the requirement for gauge-invariance of each Josephson phase.

### Second Quantization and Reduction to Maxwell-Bloch Equations

The quantization of the SCQ subsystem requires the replacement of the classical variables *φ*_*n*_ and 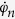 by the corresponding quantum operators 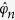 and 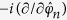, respectively. While the EM field is treated classically, the SCQs are regarded as two-level systems, so that only the two lowest energy states are retained; under these considerations, the second-quantized Hamiltonian corresponding to equation (1) is


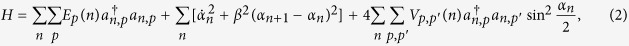


where *p, p*′ = 0, 1, *E*_0_ and *E*_1_ are the energy eigenvalues of the ground and the excited state, respectively, the operator 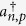 (*a*_*n*,*p*_) excites (de-excites) the *n*th SCQ from the ground to the excited (from the excited to the ground) state, and 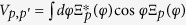 are the matrix elements of the effective SCQ-EM field interaction. The basis states Ξ_*p*_ can be obtained by solving the single-SCQ Schrödinger equation (−∂^2^/∂*φ*^2^ − *E*_*p*_ + 2 cos *φ*)Ξ_*p*_ = 0. In general, each SCQ is in a superposition state of the form 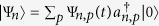. The substitution of |Ψ_*n*_〉 into the Schrödinger equation with the second-quantized Hamiltonian equation (2), and the introduction of the Bloch variables 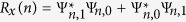, 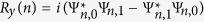, *R*_*z*_(*n*) = |Ψ_*n*,1_|^2^ − |Ψ_*n*,0_|^2^, provides the re-formulation of the problem into the Maxwell-Bloch (MB) equations


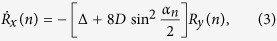



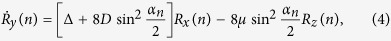



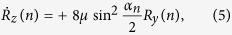


that are *nonlinearly* coupled to the resulting EM vector potential equation


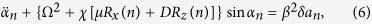


where *δα*_*n*_ = *α*_*n*−1_ − 2*α*_*n*_ + *α*_*n*+1_, *D* = (*V*_11_ − *V*_00_)/(2*χ*), Ω^2^ = (*V*_00_ + *V*_11_)/2, *μ* = *V*_10_/*χ* = *V*_01_/*χ*, and Δ = *ε*_1_ − *ε*_0_ ≡ (*E*_1_ − *E*_0_)/*χ*, with *χ* = 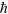
*ω*_*J*_/*E*_*J*_. In the earlier equations, the overdots denote differentiation with respect to the normalized time *t* → *ω*_*J*_*t*, in which *ω*_*J*_ = *eI*_*c*_/(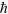
*C*) is the Josephson frequency and *e*, 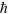 are the electron charge and the Planck’s constant devided by 2*π*, respectively.

### Approximations and Analytical Solutions

For weak EM fields, the approximation 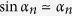 can be safely used. Then, by taking the continuum limit *α*_*n*_(*t*) → *α*(*x, t*) and *R*_*i*_(*n*; *t*) → *R*_*i*_(*x*; *t*) (*i* = *x, y, z*) of equations (3–, 16, 6), a set of simplified, yet still nonlinearly coupled equations is obtained, similar to those encountered in *two-photon* SIT in resonant media^27^. Further simplification can be achieved with the slowly varying envelope approximation (SVEA) by making for the EM vector potential the ansatz *α*(*x, t*) = *ε*(*x, t*)cos Ψ(*x, t*), where Ψ(*x, t*) = *kx* − *ωt* + *ϕ*(*x, t*) and *ε*(*x, t*), *ϕ*(*x, t*) are the slowly varying pulse envelope and phase, respectively, with *ω* and 
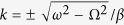
 being the frequency of the carrier wave of the EM pulse and its wavenumber in the superconducting TL, respectively. In the absence of the SCQ chain the EM pulse is “free” to propagate in the TL with speed *β*. At the same time, equations (3, 4, 5) for the Bloch vector components are transformed according to *R*_*x*_ = *r*_*x*_ cos (2Ψ) + *r*_*y*_ sin (2Ψ), *R*_*y*_ = *r*_*y*_ cos (2Ψ) − *r*_*x*_ sin (2Ψ), and *R*_*z*_ = *r*_*z*_. Then, collecting the coefficients of *sin*Ψ and *cos*Ψ while neglecting the rapidly varying terms, and averaging over the phase Ψ, results in a set of truncated equations (see Supplementary Information). Further manipulation of the resulting equations and the enforcement of the *two-photon resonance condition* Δ = 2*ω*, results in


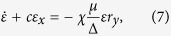



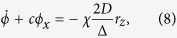


where *c* = *β*^2^*k*/*ω* = 2*β*^2^*k*/Δ, and the truncated MB equations


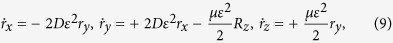


which obey the conservation law 
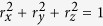
. In equation (9), the *n*–dependence of the *r*_*i*_ (*i* = *x, y, z*) is suppressed, in accordance with common practices in quantum optics.

The *r*_*i*_ can be written in terms of new Bloch vector components *S*_*i*_ using the unitary transformation *r*_*x*_ = *S*_*x*_ cos Φ − *S*_*z*_ sin Φ, *r*_*y*_ = *S*_*y*_, and *r*_*z*_ = *S*_*z*_ cos Φ + *S*_*x*_ sin Φ, where Φ is a constant angle to be determined. Using a procedure similar to that for obtaining the *r*_*i*_, we get 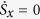, 
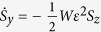
, and 
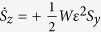
, where 
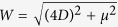
 and tan Φ ≡ *γ* = 4*D*/*μ*. The combined system of the equations for the *S*_*i*_ and equations (7, 8) admits exact solutions of the form *ε* = *ε*(*τ* = *t* − *x*/*v*) and *S*_*i*_ = *S*_*i*_(*τ* = *t* − *x*/*v*), where *v* is the pulse speed. For the slowly varying pulse envelop, we obtain


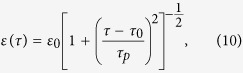


where 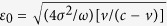 is the pulse amplitude and *τ*_*p*_ = {*χ*(*σμ*/*ω*)[*v*/(*c* − *v*)]}^−1^ its duration, with 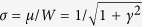. The decoherence factor *γ* can be expressed as a function of the matrix elements of the SCQ-EM field interaction, *V*_*ij*_, as *γ* = 2(*V*_11_ − *V*_00_)/*V*_10_ that can be calculated when the latter are known. Such Lorentzian propagating pulses have been obtained before in two-photon resonant media^28^,^29^; however, SIT in quantum systems has only been demonstrated in one-photon (absorbing) frequency gap media, in which solitonic pulses can propagate without dissipation^30^. The corresponding solution for the population inversion, *R*_*z*_, reads


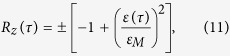


where 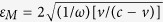, and the plus (minus) sign corresponds to absorbing (amplifying) SCQMMs; these are specified through the initial conditions as *R*_*z*_(−∞) = −1, *ε*(−∞) = 0 and *R*_*z*_(−∞ = +1), *ε*(−∞) = 0 for absorbing and amplifying SCQMMs, respectively (with *R*_*x*_(−∞) = *R*_*y*_(−∞) = 0 in both cases). The requirement for the wavenumber *k* being real, leads to the SCQ parameter-dependent condition 2*χ*^2^(*V*_11_ + *V*_00_) < (*E*_1_−*E*_0_)^2^ for pulse propagation in the SCQMM. Thus, beyond the obtained two-photon SIT or SRD, the propagating EM pulse plays a key role in the interaction processes in the qubit subsystem: it leads to collective behavior of the ensemble of SCQs in the form of quantum coherent probability pulses; such pulses are illustrated here through the population inversion *R*_*z*_.

The corresponding velocity-amplitude relation of the propagating pulse reads


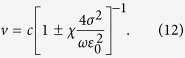


**Figure 2:**
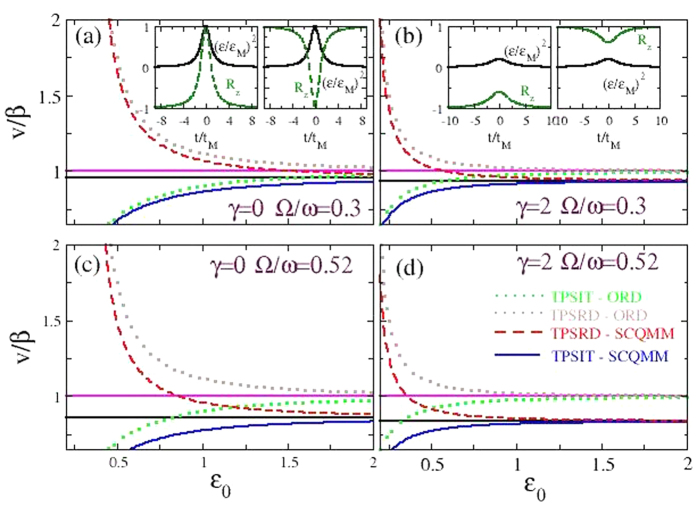
The velocity-amplitude relation in two-photon superradiant (TPSRD, amplifying) and two-photon self-induced transparent (TPSIT, absorbing) superconducting quantum metamaterials (SCQMMs) & quantum coherent pulse profiles. In all subfigures, the pulse velocity *v* in units of *β* as a function of the electromagnetic vector potential pulse amplitude *ε*_0_ is plotted and compared with the corresponding curves for ordinary (atomic) amplifying and absorbing media (brown- and green-dotted curves, respectively). The horizontal magenta-solid (resp. black-solid) lines indicate the limiting velocity in ordinary amplifying and absorbing media, *v*/*β* = 1 (resp. amplifying and absorbing SCQMMs, *v* = *c* < *β*). (**a**) *V*_00_ = *V*_11_ = 1, *V*_01_ = *V*_10_ = 0.8, *χ* = 1/5, *E*_1_ − *E*_0_ = 3 (*γ* = 0 and Ω/*ω* = 0.3). Left Inset: The electromagnetic vector potential pulse envelop (*ε*/*ε*_*M*_)^2^ and the population inversion function *R*_*z*_(*n*) profiles as a function of the slow variable (*τ*/*τ*_*M*_ in a frame of reference that is moving with velocity *v*, for TPSIT (absorbing) SCQMMs. Right Inset: Same as in the left inset for TPSRD (amplifying) SCQMMs. (**b**) *V*_00_ = 0.6, *V*_11_ = 1.4, *V*_01_ = *V*_10_ = 0.8, *χ* = 1/5, *E*_1_ − *E*_0_ = 3 (*γ* = 2 and Ω/*ω* = 0.3). Left Inset: The electromagnetic vector potential pulse envelop (*ε*/*ε*_*M*_)^2^ and the population inversion function *R*_*z*_(*n*) profiles as a function of the slow variable (*τ*/*τ*_*M*_ in a frame of reference that is moving with velocity *v*, for TPSIT (absorbing) SCQMMs in the presence of relatively strong decoherence (*γ* = 2). Right Inset: Same as in the left inset for TPSRD (amplifying) SCQMMs. (**c**) *V*_00_ = *V*_11_ = 3, *V*_01_ = *V*_10_ = 0.8, *χ* = 1/5, *E*_1_ − *E*_0_ = 3 (*γ* = 0 and Ω/*ω* = 0.52). (**d**) *V*_00_ = 3, *V*_11_ = 3.8, *V*_01_ = *V*_10_ = 0.8, *χ* = 1/5, *E*_1_ − *E*_0_ = 3 (*γ* = 2 and Ω/*ω* = 0.52). The effect of non-zero decoherence (*γ* ≠ 0) become apparent by direct comparison of (**a**) with (**b**,**c**) with (**d**). The pulse velocity *v* in SCQMMs saturates with increasing *ε*_0_ to *v*_*m*_/*β*, that can be significantly lower than that achieved in ordinary TPSIT and TPSRD media, i.e., *β*. The parameters of the SCQMM can be engineered to slow down the pulse velocity *v* at the desired level for high enough amplitudes *ε*_0_. Note that *v* is also the velocity of the coherent qubit pulse.

Equation (12) can be also written as a velocity-duration expression, since the pulse amplitude and its duration are related through 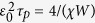. The duration of SRD pulses cannot exceed the limiting value of *τ*_*M*_ = *ω*(*c* − *v*)/(*χμv*). From equation (12), the existence of a critical velocity *c*, defined earlier, can be immediately identified; that velocity sets an upper (lower) bound on the pulse velocity in absorbing (amplifying) SCQMM structures. Thus, in absorbing (amplifying) SCQMM structures, pulses of higher intensity propagate faster (slower). That limiting velocity is generally lower than the corresponding one for two-photon SIT or SRD in ordinary media, *β*, which here coincides with the speed of the “free” pulse in the TL (Fig. 2). As can be inferred from Fig. 2, the increase of decoherence through *γ* makes the velocity to saturate at its limiting value *c* at lower amplitudes *ε*; that velocity can be reduced further with increasing the ratio of the TL to the pulse carrier wave frequency Ω/*ω* through proper parameter engineering. Moreover, effective control of *v* in SCQMMs could in principle be achieved by an external field^31^ or by real time tuning of the qubit parameters. That ability to control the flow of “optical”, in the broad sense, information may have technological relevance to quantum computing^25^. Note that total inversion, i.e. excitation or de-excitation of all qubits during pulse propagation is possible only if *γ* = 0, i.e., for *V*_00_ = *V*_11_; otherwise (*V*_00_ < *V*_11_) the energy levels of the qubit states are Stark-shifted, violating thus the resonance condition. Typical analytical profiles for the EM vector potential pulse *ε*(*τ*) and the population inversions *R*_*z*_(*τ*) both for absorbing and amplifying SCQMMs are shown in the insets of Fig. 2. The maximum of *ε*(*τ*) reduces considerably with increasing *γ*, while at the same time the maximum (minimum) of *R*_*z*_ decreases (increases) at the same rate.

The system of equations (7, 25–9) can be reduced to a single equation using the parametrization *r*_*x*_ = *R*_0_*γσ*^2^[1 − cos *θ*], *r*_*y*_ = −*R*_0_*σ *sin *θ*, and *r*_*z*_ = *R*_0_{1 − *σ*^2^[1 − cos *θ*]}, of the Bloch vector components. Then, a relation between the Bloch angle *θ* ≡ *θ*(*x, t*) and the slow amplitude *ε* can be easily obtained, that leads straightforwardly to the equation 
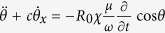
. Time integration of that equation yields 
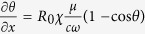
, that conforms with the famous *area theorem: pulses with special values of* “*area*” *θ*(*x*) = 2*πn conserve that value during propagation*.

Here we concentrate on the interaction of the SCQs with the EM wave and we are not concerned with decoherence effects in the SCQs due to dephasing and energy relaxation. This is clearly an idealization which is justified as long as the coherence time exceeds the wave propagation time across a relatively large number of unit cell periods. In a recent experiment^26^, a charge qubit coupled to a strip line had a dephasing time in excess of 200 *ns*, i.e., a dephasing rate of 5 *MHz*, and a photon loss rate from the cavity of 0.57 *MHz*. Those frequencies are very small compared with the transition frequency of the considered SCQs which is of the order of the Josephson energy (i.e., a few *GHz*)^17^,^18^. Therefore, we have neglected such decoherence effects in the present work. The decoherence factor *γ*, which in Fig. 2b,d has been chosen according to the parameter values in ref. 17, is not related to either dephasing or energy relaxation. That factor attains a non-zero value whenever the matrix elements of the effective SCQ-EM field interaction, *V*_11_ and *V*_00_, are not equal.

### Numerical Simulations

**Figure 3:**
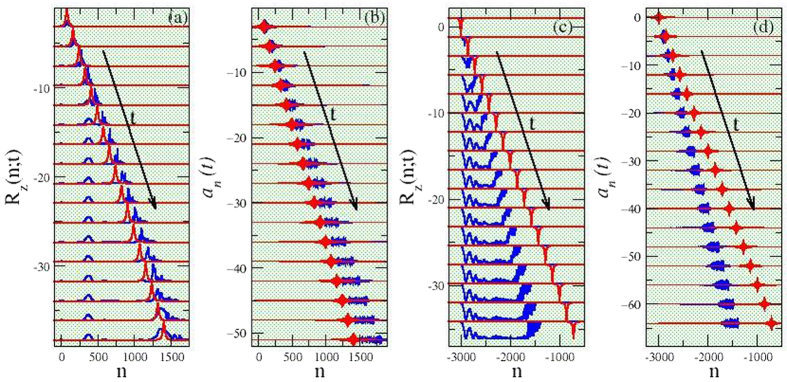
Numerical validation of the analytical expressions for two-photon self-induced transparent (TPSIT) and superradiant (TPSRD) propagating pulses. (**a**) Snapshots of the population inversion pulse *R*_*z*_(*n*; *t*), excited by the induced quantum coherence in the qubit subsystem by the electromagnetic vector potential pulse, in the absence of decoherence (*γ* = 0); the pulse propagates to the right (time increases downwards) in TPSIT (absorbing) superconducting quantum metamaterials (SCQMMs). The snapshots are taken at intervals of 20 time-units starting at *t* = 20 and they are displaced vertically to avoid overlapping (blue pulses). The corresponding pulses from the analytical expression equation (11) at the same time-instants are shown in red. (**b**) Snapshots for the corresponding evolution of the electromagnetic vector potential pulse *a*_*n*_(*t*), that exhibits significant broadening as time passes by; the numerical and analytical pulses are shown in blue and red color, respectively. (**c**) The same as in a in TPSRD (amplifying) superconducting quantum metamaterials. The resulting propagation is not as simple as expected from the theoretical analysis; instead of a population inversion pulse, it is observed a rather kink-like front propagating to the the right (blue) with a velocity considerably less than that predicted analytically for the pulse, which analytical form is shown in red. (**d**) The same as in (**b**) in TPSRD (amplifying) superconducting quantum metamaterials. The velocity of the *a*_*n*_(*t*) pulse (blue) is the same as that of the propagating population inversion front, *R*_*z*_(*n*; *t*); however, it exhibits less broadening with time in comparison with the corresponding numerical *a*_*n*_(*t*) pulse in b. The predicted analytical form is shown in red. Parameter values: *χ* = 1/5, *β* = 6, *V*_00_ = *V*_11_ = 1, *V*_01_ = *V*_10_ = 0.8, *E*_1_ − *E*_0_ = 3, and *v*/*c* = 0.7 (for (**a**,**b**)); *v*/*c* = 1.25 (for (**c**,**d**)).

In order to confirm numerically the obtained results, the equations (3–, 16, 6) are integrated in time using a fourth order Runge-Kutta algorithm with constant time-step. For pulse propagation in absorbing SCQMMs, all the qubits are initially set to their ground state while the vector potential pulse assumes its analytical form for the given set of parameters. A very fine time-step and very large qubit arrays are used to diminish the energy and/or probability loss and the effects of the boundaries during propagation, respectively. The subsequent temporal evolution in two-photon SIT SCQMM, as can be seen in Fig. 3a,b, in which several snapshots of the population inversion *R*_*z*_(*n*; *t*) and the vector potential pulses *a*_*n*_(*t*), respectively, are shown, reveals that the latter are indeed capable of inducing quantum coherent effects in the qubit subsystem in the form of population inversion pulses! In Fig. 3a, the amplitude of the *R*_*z*_(*n*; *t*) pulse gradually grow to the expected maximum around unity in approximately 60 time units, and they continue its course almost coherently (although with fluctuating amplitude) for about 160 more time units, during which they move at the same speed as the vector potential pulse (Fig. 3b). However, due to the inherent discreteness in the qubit subsystem and the lack of inter-qubit coupling, the *R*_*z*_(*n*; *t*) pulse splits at certain instants leaving behind small “probability bumps” that get pinned at particular qubits. After the end of the almost coherent propagation regime, the *R*_*z*_(*n*; *t*) pulse broadens and slows-down until it stops completely. At the same time, the width of the *a*_*n*_(*t*) pulse increases in the course of time due to discreteness-induced dispersion. A comparison with the corresponding analytical expressions reveals fair agreement during the almost coherent propagation regime, although both the *R*_*z*_(*n*; *t*) and *a*_*n*_(*t*) pulses travel slightly faster than expected from the analytical predictions. The temporal variable here is normalized to the inverse of the Josephson frequency *ω*_*J*_ which for typical parameter values is of the order of a few *GHz*^17^. Then, the almost coherent induced pulse regime in the particular case shown in Fig. 3 lasts for ~160 × 10^−9^ s, or ~160 *ns*, which is of the same order as the reported decoherence time for a charge qubit in ref. 26 (i.e., 200 *ns*).

The situation seems to be different, however, in the case of two-photon SRD pulses, as can be observed in the snapshots shown in Fig. 3c,d for *R*_*z*_(*n*; *t*) and *a*_*n*_(*t*), respectively. Here, the lack of the inter-qubit interaction is crucial, since the SCQs that make a transition from the excited to their ground state as the peak of the *a*_*n*_(*t*) pulse passes by their location, cannot return to their excited states after the *a*_*n*_(*t*) pulse has gone away. It seems, thus, that the *a*_*n*_(*t*) pulse creates a type of a kink-like front that propagates at the same velocity. It should be noted that the common velocity of the *R*_*z*_(*n*; *t*) and *a*_*n*_(*t*) pulses is considerably different (i.e., smaller) than the analytically predicted one, as it can be inferred by inspection of Fig. 3c,d. Even more complicated behavioral patterns of two-photon SRD propagating pulses and the effect of non-zero decoherence factor are discussed in the Supplementary Information.

## Conclusion

An SCQMM comprising SCQs loaded periodically on a superconducting TL has been investigated theoretically using a minimalistic one-dimensional model following a semiclassical approach. While the SCQs are regarded as two-level quantum systems, the EM field is treated classically. Through analytical techniques it is demonstrated that the system allows self-induced transparent and superradiant pulse propagation given that a particular constraint is fulfilled. Most importanty, it is demonstrated that the propagating EM pulses may induce quantum coherent population inversion pulses in the SCQMM. Numerical simulation of the semiclassical equations confirms the excitation of population inversion pulses with significant coherence time in absorbing media. The situation is slightly different in amplifying media, in which the numerically obtained, induced population inversion excitations are kink-like propagating structures (although more complex behaviors discussed in the Supplementary Information also appear). Moreover, the limiting pulse velocity in both amplifying and absorbing SCQMMs is lower than the corresponding one in two-photon resonant amplifying and absorbing ordinanary (atomic) media. That limiting velocity in SCQMMs can in principle be engineered through the SCQ parameters.

## Additional Information

**How to cite this article**: Ivić, Z. *et al*. Qubit lattice coherence induced by electromagnetic pulses in superconducting metamaterials. *Sci. Rep.* **6**, 29374; doi: 10.1038/srep29374 (2016).

## Supplementary Material

Supplementary Information
