# Supplementary material for: Qubit lattice coherence induced by electromagnetic pulses in superconducting metamaterials
Source: Sci Rep. 2016 Jul 12;6:29374. doi: 10.1038/srep29374 (PMC4941529; doi:10.1038/srep29374)
Supplement: Supplementary Information [file srep29374-s1.pdf]

Supplementary Information: Qubit lattice coherence induced by electromagnetic pulses in superconducting metamaterials

Z. Ivić[1,2,5], N. Lazarides[1,3,5], G. P. Tsironis[1,3,4,5]
[1] *Crete Center for Quantum Complexity and Nanotechnology, Department of Physics, University of Crete, P. O. Box 2208, 71003 Heraklion, Greece;*
[2] *University of Belgrade, "Vinča" Institute of Nuclear Sciences, Laboratory for Theoretical and Condensed Matter Physics, P.O.Box 522, 11001 Belgrade, Serbia*
[3] *Institute of Electronic Structure and Laser, Foundation for Research and Technology–Hellas, P.O. Box 1527, 71110 Heraklion, Greece*
[4] *Department of Physics, School of Science and Technology, Nazarbayev University, 53 Kabanbay Batyr Ave., Astana 010000, Kazakhstan*
[5] *National University of Science and Technology MISiS, Leninsky prosp. 4, Moscow, 119049, Russia*
(Dated: December 4, 2015)

I. HAMILTONIAN FUNCTION AND QUANTIZATION OF THE QUBIT SUBSYSTEM

Consider an infinite number of superconducting charge qubits in a transmission line (TL) that consists of two superconducting plates separated by distance d, while the center-to-center distance between qubits, ℓ, is of the same order of magnitude. The charge qubit is of the form of a mesoscopic superconducting island which is connected to each electrode of the TL with a Josephson junction (JJ). Assume that an electromagnetic (EM) wave corresponding to a vector potential $\vec{A} = A_z(x,t)\hat{z}$ propagates along the superconducting TL, in a direction parallel to the superconducting electrodes and perpendicular to the direction of the EM wave propagation. A minimalistic description of that system constists of writing the Hamiltonian of the compound qubit array - EM field system with the geometry shown in Fig. 1 of the paper, as

$$H = \sum_n \left\{ \dot{\varphi}_n^2 - 2\cos\alpha_n \cos\varphi_n + \dot{\alpha}_n^2 + \beta^2(\alpha_{n+1} - \alpha_n)^2 \right\}, \tag{1}$$

where φ_n is the superconducting phase on $n-$th island, $\beta^2 = (8\pi d E_J)^{-1} \left(\Phi_0/(2\pi)\right)^2$, with d being the separation between the superconducting electrodes of the TL, and the overdots denote derivation with respect to the temporal variable t. In what follows, a basic assumption is that the wavelength λ of the EM field is much larger than the other length scales determined by the size of the unit cell of the qubit metamaterial ($\lambda >> \ell, d$), so that the vector potential component $A_z(x,t)$ can be regarded to be approximately constant within a unit cell, $A_z(x,t) \simeq A_{z,n}(t)$. Then, the discretized and normalized EM vector potential at the $n-$th unit cell which appears in Eq. (1) reads $a_n(t) = (2\pi d/\Phi_0) A_n(t)$. The Hamiltonian function Eq. (1) is given in units of the Josephson energy $E_J = (\Phi_0 I_c)/(2\pi C)$, where I_c and C is the critical current and capacitance, respectively, of the JJs, and $\Phi_0 = h/(2e)$ is the flux quantum, with h and e being the Planck's constant and the electron charge, respectively. That Hamiltonian can be written in a more transparent form by adding and subtracting $2\cos\phi_n$ and subsequently rearranging to get

$$H = H_{QUB} + H_{EMF} + H_{int}, \tag{2}$$

where the qubit subsystem energy H_{QUB}, the EM field energy H_{EMF}, and their interaction energy H_{int}, take respectively the form

$$H_{QUB} = \sum_n \{\dot{\varphi}_n^2 - 2\cos\varphi_n\}, \qquad H_{EMF} = \sum_n \{\dot{\alpha}_n^2 + \beta^2(\alpha_{n+1} - \alpha_n)^2\}, \qquad H_{int} = \sum_n \{2\cos\varphi_n (1 - \cos\alpha_n)\}. \tag{3}$$

In the following, the EM field is treated classically, while the qubits are regarded as two-level systems. The latter approximation is particularly well suited in the case of *resonant qubit - EM field interaction* adopted here.

The quantization of the qubit subsystem can be formally performed by replacing the classical variables φ_n and $\dot{\varphi}_n$ by the quantum operators $\hat{\varphi_n}$ and $\dot{\varphi}_n \rightarrow -i\frac{\partial}{\partial\varphi_n}$, respectively, in the Hamiltonian H_{QUB} since we are dealing with a large number of Cooper pairs. The exact energy spectrum $E_p(n)$ and the corresponding wavefunctions $\Xi_p(n)$ of the nth qubit may then be obtained by mapping the Schrödinger equation with the single-qubit Hamiltonian $H_{sq} = \dot{\varphi}_n^2 - 2\cos\varphi_n$, onto the Mathieu equation

$$\left(\frac{\partial^2}{\partial\varphi_n^2} + E_{p,n} - 2\cos\varphi_n \right) \Xi_{p,n} = 0. \tag{4}$$

The second quantization of the qubit subsystem proceeds by rewritting the single-qubit Hamiltonian as

$$H_{sq} \rightarrow H_{sq} = -\int d\varphi_n \hat{\Psi}^\dagger(\varphi)\left(\frac{\partial^2}{\partial\varphi^2} + 2\cos\varphi\right)\hat{\Psi}(\varphi), \tag{5}$$

where $\hat{\Psi}^\dagger$ and $\hat{\Psi}$ are field operators. Note that the subscript "n" in Eq. (5) has been dropped since the qubits are identical. Using the expansion $\hat{\Psi}(\varphi) = \sum_p a_p \Xi_p(\varphi)$, where the operators $a_p^\dagger$ (a_p) create (annihilate) qubit excitations of energy E_p, the Hamiltonian Eq. (5) is transformed into

$$H_{sq} = \sum_{p=0,1,...} E_p a_p^\dagger a_p. \tag{6}$$

We hereafter restrict H_{sq} to the Hilbert subspace of its two lowest levels, i.e., those with $p = 0, 1$, so that in second quantized form the Hamiltonian Eq. (2) reads

$$H = \sum_n \sum_p E_p(n) a_{n,p}^\dagger a_{n,p} + \sum_{p,p'} V_{p,p'}(n) a_{n,p}^\dagger a_{n,p'} \sin^2\frac{\alpha_n}{2} + \sum_n \{\dot{\alpha}_n^2 + \beta^2(\alpha_{n+1} - \alpha_n)^2\}, \tag{7}$$

where $p, p' = 0, 1$ and the $V_{p,p'}(n) \equiv V_{p',p}(n)$ that represent the matrix elements of the nth qubit - EM field interaction, are given by

$$V_{p,p'}(n) = \int d\varphi_n \Xi_p^*(\varphi_n) \cos\varphi_n \Xi_{p,n}(\varphi_n). \tag{8}$$

In the reduced state space, in which a single qubit can be either in the ground ($p = 0$) or in the excited ($p = 1$) state, the normalization condition $\sum_p a_{n,p}^\dagger a_{n,p} = 1$ holds for each qubit in the metamaterial. The reduced Hamiltonian Eq. (7) is also written in units of E_J.

II. MAXWELL-BLOCH FORMULATION OF THE DYNAMIC EQUATIONS

In accordance with the adopted semiclassical approach, the time-dependent Schrödinger equation

$$i\hbar\frac{\partial}{\partial t}|\Psi\rangle = \bar{H}|\Psi\rangle, \tag{9}$$

in which $\bar{H}$ is the Hamiltonian from Eq. (7) in physical units, i.e., $\bar{H} = HE_J$, is employed for the description of the qubit subsystem. Generally, the state of each qubit is a superposition of the form

$$|\Psi_n\rangle = \sum_p \Psi_{n,p}(t) a_{n,p}^\dagger |0\rangle, \tag{10}$$

for which it can be easily shown that the coefficients $\Psi_{n,p}$ satisfy the following normalization conditions

$$\sum_p |\Psi_{n,p}(t)|^2 = 1, \qquad \sum_{n,p} |\Psi_{n,p}(t)|^2 = N, \tag{11}$$

where in the second Eqs. (11) a finite $N-$qubit subsystem has been implied. Assuming that the pulse power is not very strong, substantial simplification of the dynamic equations for the qubit subsystem can be achieved using the approximation $[1 - \cos(\alpha_n)] \simeq (1/2)\alpha_n^2$ in the interaction Hamiltonian H_{int}. Substitution of $|\Psi\rangle = |\Psi_n\rangle$ from Eq. (10) into the Schrödinger equation (9), along with derivation of the classical Hamilton's equation for the normalized EM vector potential α_n, yields

$$i\dot{\Psi}_{n,p} = \epsilon_p \Psi_{n,p} + \frac{1}{\chi}\sum_{p'} V_{p,p'}(n)\Psi_{n,p'}\alpha_n^2, \tag{12}$$

$$\ddot{\alpha}_n - \beta^2(\alpha_{n+1} + \alpha_{n-1} - 2\alpha_n) + \sum_{p,p'} V_{p,p'}\Psi_{n,p}^*\Psi_{n,p'}\alpha_n = 0, \tag{13}$$

where $\chi = \hbar\omega_j/E_J$. In Eqs. (12) and (13), the temporal variable is renormalized according to $t \rightarrow \omega_J t$. Because of that, the dimensionless energy of the qubit excitations has to be redefined according to $E_P \rightarrow \epsilon_p = E_p/\chi$, which explains the presence of the dimensionless factor $1/\chi$ in Eq. (12).

The evolution Eqs. (12) and (13) can be rewritten in terms of the $n-$dependent Bloch vector components through the transformation

$$R_z(n) = |\Psi_{n,1}|^2 - |\Psi_{n,0}|^2, \qquad R_y(n) = i(\Psi^*_{n,0}\Psi_{n,1} - \Psi^*_{n,1}\Psi_{n,0}), \qquad R_x(n) = \Psi^*_{n,1}\Psi_{n,0} + \Psi^*_{n,0}\Psi_{n,1}. \tag{14}$$

in which the variables R_i $(i = x, y, z)$ apply to each single qubit. Thus, from the point of view of a macroscopic system, $|\Psi_i|^2$ represent the population densities $\varrho_i = \frac{N}{N}|\Psi|^2 \equiv \frac{N_i}{N}$. By transforming Eqs. (12) and (13) according to Eq. (14) we get

$$\dot{R}_x(n) = -(\Delta + 2D\alpha_n^2)R_y(n), \qquad \dot{R}_y(n) = +(\Delta + 2D\alpha_n^2)R_x(n) - 2\mu\alpha_n^2 R_z(n), \qquad \dot{R}_z(n) = +2\mu\alpha_n^2 R_y(n), \tag{15}$$

$$\ddot{\alpha}_n + \chi[\Omega^2 + \mu R_x(n) + DR_z(n)]\alpha_n = \beta^2(\alpha_{n-1} - 2\alpha_n + \alpha_{n+1}), \tag{16}$$

where

$$D = \frac{(V_{11} - V_{00})}{2\chi}, \qquad \Omega^2 = \frac{(V_{11} + V_{00})}{2}, \qquad \mu = \frac{V_{10}}{\chi}, \qquad \Delta = \epsilon_1 - \epsilon_0 \equiv \frac{(E_1 - E_0)}{\chi}. \tag{17}$$

By taking the continuous limit of Eqs. (15) and (16) using the relations $\alpha_n \to \alpha(x,t)$ and $\alpha_{n\pm1} \approx \alpha \pm \alpha_x + \frac{1}{2}\alpha_{xx}$, we obtain

$$\dot{R}_x = -(\Delta + 2D\alpha^2)R_y, \qquad \dot{R}_y = +(\Delta + 2D\alpha^2)R_x - 2\mu\alpha^2 R_z, \qquad \dot{R}_z = +2\mu\alpha^2 R_y, \tag{18}$$

$$\ddot{\alpha} - \beta^2\alpha_{xx} + \Omega^2\alpha = -\chi(DR_z + \mu R_x)\alpha, \tag{19}$$

where R_x, R_y, R_z, and α are functions of the spatial variable x and normalized temporal variable t, while the overdots denote partial derivation with respect to the latter. It can be easily verified that the Bloch equations Eqs. (18) possess the dynamic invariant $\sum_i R_i^2 = 1$, whose constant value is derived through the normalization conditions.

III. SLOWLY VARYING ENVELOPE APPROXIMATION (SVEA)

The Slowly Varying Envelope Approximation (SVEA) relies on the assumption that the envelop of a travelling pulse in a nonlinear medium varies slowly in both time and space compared with the period of the carrier wave, that makes possible the introduction of slow and fast variables. Assuming that in the following, we can approximate the EM vector potential function by

$$\alpha(x,t) = \varepsilon(x,t)\cos\psi(x,t), \tag{20}$$

where $\psi(x,t) = kx - \omega t + \phi(x,t)$, with k and ω being the wavenumber and frequency of the carrier wave, respectively, that are connected through the dispersion relation that is obtained at a later stage. The functions $\varepsilon(x,t)$ and $\phi(x,t)$ in Eq. (20) are the slowly varying envelop and phase, respectively. Using fast and slow variables, the x and y Bloch vector components, $R_x(n)$ and $R_y(n)$, can be expressed as a function of new, in-phase and out-of-phase Bloch vector components r_x and r_y as

$$R_x = r_x\cos(2\psi) + r_y\sin(2\psi), \qquad R_y = r_y\cos(2\psi) - r_x\sin(2\psi), \qquad R_z = r_z. \tag{21}$$

A. Derivation of the dynamic equations for the slowly varying envelop and phase of the electromagnetic vector potential

From Eqs. (20) and (21), the second temporal and spatial derivative of $\alpha(x,t)$ can be approximated by

$$\ddot{\alpha} \approx 2\omega\dot{\varepsilon}\sin\psi + (2\omega\dot{\phi} - \omega^2)\varepsilon\cos\psi, \qquad \alpha_{xx} \approx -2k\varepsilon_x\sin\psi - (2k\phi_x - k^2)\varepsilon\cos\psi, \tag{22}$$

in which the rapidly varying terms of the form $\ddot{\varepsilon}$, ε_{xx}, ϕ^2, ϕ_{xx}, $\ddot{\phi}$, $\phi_x\varepsilon_x$, etc., have been neglected. Substitution of Eqs. (22) and (21), into Eq. (19) gives

$$2(\omega\dot{\varepsilon} + k\beta^2\varepsilon_x)\sin\psi + [2(\dot{\phi}\omega + k\phi_x) - \omega^2 + \Omega^2 + \beta^2k^2]\varepsilon\cos\psi = -\chi\{Dr_z + \mu[r_x\cos(2\psi) + r_y\sin(2\psi)]\}\varepsilon\cos\psi. \tag{23}$$

Equating the coefficients of $\sin\psi$ and $\cos\psi$ in the earlier equation yields

$$\omega\dot{\varepsilon} + k\beta^2\varepsilon_x = -\chi\mu r_y\varepsilon\cos^2\psi, \tag{24}$$

and

$$2(\dot{\phi}\omega + k\phi_x) - \{\omega^2 - \Omega^2 - \beta^2 k^2\} = -\chi[Dr_z + \mu r_x \cos(2\psi)]. \tag{25}$$

In order to obtain the dispersion relation $\omega = \omega(k)$, we require the vanishing of the expression in the curly brackets in Eq. (25), which yields for the wavevector of the EM radiation (i.e., the pulse) in the TL the expression

$$k = \pm \frac{\sqrt{\omega^2 - \Omega^2}}{\beta}. \tag{26}$$

Thus, the EM wave can propagate through the superconducting quantum metamaterial only when its frequency exceeds a critical one, $\omega_c = \Omega = \sqrt{(V_{00} + V_{11})/2}$. Finally, Eqs. (24) and (25) are averaged in time over the period $T = 2\pi/\omega$ (i.e., the fast time-scale) of the phase $\psi(x,t)$. Due to the simple time-dependence of $\psi(x,t)$ that has been assumed earlier in the framework of SVEA, that averaging actually requires the calculation of integrals of the form

$$\langle \mathcal{F}(\sin f(\psi), \cos g(\psi)) \rangle = \frac{1}{2\pi} \int_0^{2\pi} \mathcal{F}(\sin f(\psi), \cos g(\psi)) d\psi.$$

This procedure, when it is applied to the two evolution equations Eqs. (24) and (25) gives the truncated equations for slow amplitude and phase

$$\dot{\varepsilon} + c\varepsilon_x = -\chi \frac{\mu}{2\omega} \varepsilon r_y, \qquad \dot{\phi} + c\phi_x = -\chi \frac{D}{\omega} R_z, \tag{27}$$

where $c = \beta^2 k/\omega$ is a critical velocity.

B. Derivation of dynamic equations for the transformed Bloch vector components

Substituting Eqs. (20) and (21) into Eqs. (18) for the original Bloch vector components, we get

$$(\dot{r}_x + 2\dot{\psi} r_y)\cos(2\psi) + (\dot{r}_y - 2\dot{\psi} r_x)\sin(2\psi) = -(\Delta + 2D\varepsilon^2 \cos^2\psi)[r_y \cos(2\psi) - r_x \sin(2\psi)] \tag{28}$$

$$(\dot{r}_y - 2\dot{\psi} r_x)\cos(2\psi) - (\dot{r}_x + 2\dot{\psi} r_y)\sin(2\psi) = (\Delta + 2D\varepsilon^2 \cos^2\psi)[r_x \cos(2\psi) + r_y \sin(2\psi)] - 2\mu\varepsilon^2 \cos^2\psi r_z \tag{29}$$

$$\dot{r}_z = 2\mu\varepsilon^2 \cos^2\psi [r_y \cos(2\psi) - r_x \sin(2\psi)]. \tag{30}$$

In order to derive the dynamic equations for the new Bloch vector components r_i, we first multiply Eqs. (28) and (29) by $\cos(2\psi)$ and $\sin(2\psi)$, respectively, and then subtract one equation from the other. Thus we get

$$\dot{r}_x + 2\dot{\psi} r_y = -(\Delta + 2D\varepsilon^2 \cos^2\psi) r_y + 2\mu\varepsilon^2 \cos^2\psi \cos(2\psi) r_z. \tag{31}$$

Similarly, by multiplying Eqs. (28) and (29) by $\sin(2\psi)$ and $\cos(2\psi)$, respectively, and by adding them, we get

$$\dot{r}_y - 2\dot{\psi} r_x = (\Delta + 2D\varepsilon^2 \cos^2\psi) r_x - 2\mu\varepsilon^2 \cos^2\psi \sin(2\psi) r_z. \tag{32}$$

Finally, an average over the phase ψ is performed onto Eqs. (30)-(32) using the (easy to prove) relations $\langle \cos^2\psi \cos(2\psi) \rangle = 1/4$ and $\langle \cos^2\psi \sin(2\psi) \rangle = 0$. That procedure, and using also the relation $\dot{\psi} = \dot{\phi} - \omega$, yields the truncated Bloch equations

$$\dot{r}_x = -(\delta + 2\dot{\phi} + D\varepsilon^2) r_y - \frac{\mu}{2}\varepsilon^2 r_z, \qquad \dot{r}_y = +(\delta + 2\dot{\phi} + D\varepsilon^2) r_x, \qquad \dot{r}_z = \frac{\mu}{2}\varepsilon^2 r_y, \tag{33}$$

where $\delta = \Delta - 2\omega$. Note that Eqs. (33) possess a dynamic invariant that has a form similar to that of the original Bloch equations, Eqs. (18), i.e.,

$$r_x^2 + r_y^2 + r_z^2 = 1. \tag{34}$$

The truncated Bloch equations Eqs. (33), along with Eqs. (27) for the slowly varying amplitude and phase of the EM vector potential pulse, constitute a closed system with five unknown functions which describe the approximate dynamics of the superconducting quantum metamaterial that are determined in the next section.

IV. EXACT INTEGRATION OF THE TRUNCATED EQUATIONS

The combination of Eqs. (27) and the third equation of Eqs. (33) provides a relation between the slow amplitude and the phase of the EM vector potential pulse. The first of Eqs. (27) is multiplied by ε and written as

$$\left[\frac{\partial}{\partial t}+c\frac{\partial}{\partial x}\right]\varepsilon^2(x,t)=-\chi\frac{\mu}{\omega}\varepsilon^2(x,t)r_y, \tag{35}$$

while the derivative of the second of Eqs. (27) is taken with respect to time and then $\dot{R}_z=\dot{r}_z$ is replaced from Eqs. (33). Thus we get

$$\left[\frac{\partial}{\partial t}+c\frac{\partial}{\partial x}\right]\dot{\phi}(x,t)=-\chi\frac{\mu D}{2\omega}\varepsilon^2(x,t)r_y. \tag{36}$$

From Eqs. (35) and (36), and by taking into account the independence of the slow temporal and spatial variables, we get

$$2\dot{\phi}(x,t)=D\varepsilon^2(x,t)+const., \tag{37}$$

where the constant of integration can be set equal to zero. Using Eq. (37), the truncated Bloch equations Eqs. (33) can be written as

$$\dot{r}_x=-(\delta+2D\varepsilon^2)r_y, \qquad \dot{r}_y=+(\delta+2D\varepsilon^2)r_x-\frac{\mu}{2}\varepsilon^2r_z, \qquad \dot{r}_z=\frac{\mu}{2}\varepsilon^2r_y. \tag{38}$$

The latter can be written in a simpler form using the unitary transformation

$$r_x=S_x\cos\Phi-S_z\sin\Phi, \qquad r_y=S_y, \qquad r_z=S_z\cos\Phi+S_x\sin\Phi, \tag{39}$$

where Φ is the constant transformation angle that is going to be determined. The truncated Bloch equations for the r_i, $i=x,y,z$, can be written in terms of the new Bloch vector components S_i, using a procedure similar to that used in Subsection III.B to obtain Eqs. (33). Sustituting Eqs. (39) into Eqs. (38), we get

$$\dot{S}_x\cos\Phi-\dot{S}_z\sin\phi=-(\delta+2D\varepsilon^2)S_y, \tag{40}$$

$$\dot{S}_y=\left[(\delta+2D\varepsilon^2)\cos\Phi-\frac{\mu}{2}\varepsilon^2\sin\Phi\right]S_x-\left[(\delta+2D\varepsilon^2)\sin\Phi+\frac{\mu}{2}\varepsilon^2\cos\Phi\right]S_z, \tag{41}$$

$$\dot{S}_x\sin\Phi+\dot{S}_z\cos\Phi=-\frac{\mu}{2}\varepsilon^2S_y. \tag{42}$$

Multiplying Eqs. (40) and (42) by $\cos\Phi$ and $\sin\Phi$, respectively, and then adding them together, we get

$$\dot{S}_x=\left\{\varepsilon^2\left[\frac{1}{2}\mu\sin\Phi-2D\cos\Phi\right]-\delta\cos\Phi\right\}S_y. \tag{43}$$

Similarly, multiplying Eqs. (40) and (42) by $\sin\Phi$ and $\cos\Phi$, respectively, and then subtracting one equation from the other, we get

$$\dot{S}_z=\left\{\varepsilon^2\left[\frac{1}{2}\mu\cos\Phi+2D\sin\Phi\right]-\delta\sin\Phi\right\}S_y. \tag{44}$$

The transformation angle Φ can now be selected so that the resulting equations are simplified as much as possible. We thus define

$$\tan\Phi\equiv\gamma=\frac{4D}{\mu}, \tag{45}$$

so that $\cos\Phi=\pm\sigma$ and $\sin\Phi=\pm\sigma\gamma$ where $\sigma=1/\sqrt{1+\gamma^2}$. The choice of the sign is irrelevant and here we pick positive sign for both functions. Using the above value for the transformation angle and the definitions $W=\sqrt{(4D)^2+\mu^2}$ and $\eta=-\delta\mu/W$, Eqs. (43), (41), and (44) obtain their final form

$$\dot{S}_x=+\eta S_y, \qquad \dot{S}_y=-\eta S_x+\left[\eta\gamma-\frac{1}{2}W\varepsilon^2\right]S_z, \qquad \dot{S}_z=\left[-\eta\gamma+\frac{1}{2}W\varepsilon^2\right]S_y. \tag{46}$$

In order to investigate the possibility for "coherent propagation" of an electromagnetic potential pulse, we consider resonance conditions. In that case, $\eta = 0$, and Eqs. (46) become

$$\dot{S}_x = 0, \qquad \dot{S}_y = -\frac{1}{2}W\varepsilon^2 S_z, \qquad \dot{S}_z = +\frac{1}{2}W\varepsilon^2 S_y. \tag{47}$$

Combining the second and third Eq. (47) and integrating, we obtain the "resonant" conservation law $S_y^2 + S_z^2 = const.$. Assuming that all the qubits are in the ground state at $t = -\infty$, we have the initial conditions $r_x(t = -\infty) = r_y(t = -\infty) = 0$ and $r_z(t = -\infty) = -1$ which are transformed into $S_x(t = -\infty) = -\gamma\sigma$, $S_y(t = -\infty) = 0$, and $S_z(t = -\infty) = -\sigma$ through the transformation Eq. (39). Applying the initial conditions to the resonant conservation law, we get

$$S_y^2 + S_z^2 = \sigma^2. \tag{48}$$

In the following, we seek solution of the form $\varepsilon = \varepsilon(\tau = t - x/v)$ and $S_i = S_i(\tau = t - x/v)$, with $i = x, y, z$. By changing the variables in the first of Eqs. (27), with r_y being replaced by S_y, we get after rearramgement

$$\frac{\varepsilon_\tau}{\varepsilon} = \chi \frac{\mu}{2\omega} \frac{v}{c - v} S_y. \tag{49}$$

Then, combining Eq. (29) with the third Eq. (47) and integrating, we get

$$\varepsilon^2(\tau) = \chi \frac{2\mu}{\omega W} \frac{v}{c - v} [S_z(\tau) + \sigma], \tag{50}$$

where the conditions $\varepsilon(-\infty)$ and $S_z(-\infty) = -\sigma$ were used. The system of Eqs. (48)-(50) for ε, S_y, and S_z can be integrated exactly; the variables S_y and S_z can be eliminated in favour of ε to give $\varepsilon_\tau = \lambda\varepsilon^2\sqrt{a + b\varepsilon^2}$, in which the constants are defined as $a = 2\sigma/\kappa$, $b = -1/\kappa^2$, $\lambda = \chi\frac{\mu}{2\omega}\frac{v}{c-v}$, $\kappa = \frac{2\mu}{\omega W}\frac{v}{c-v}$ to simplify the notation. The equation for ε can be readily integrated

$$\int_{\varepsilon_0}^{\varepsilon} \frac{d\varepsilon}{\varepsilon^2\sqrt{a + b\varepsilon^2}} = \lambda \int_{\tau_0}^{\tau} d\tau \Rightarrow -\frac{\sqrt{a + b\varepsilon^2}}{a\varepsilon} = \lambda(\tau - \tau_0), \tag{51}$$

where we have set $\varepsilon_0 \equiv \varepsilon(\tau = \tau_0) = \sqrt{2\sigma\kappa}$ to eliminate the boundary term resulting from the integral over ε. Solving Eq. (51) for ε, we finally get a Lorentzian-like slowly varying amplitude

$$\varepsilon(\tau) = \frac{\varepsilon_0}{\sqrt{1 + \tau_p^{-2}(\tau - \tau_0)^2}}, \tag{52}$$

where $\varepsilon_0 = \sqrt{-a/b} = \sqrt{2\sigma\kappa}$ and $\tau_p^{-2} = -\frac{a^2\lambda^2}{b} = \left(\chi\frac{\sigma\mu}{\omega}\right)^2 \left(\frac{v}{c-v}\right)^2$.

V. DETAILS OF THE NUMERICAL SIMULATIONS AND THE ROLE OF DECOHERENCE

The numerical simulations in order to confirm (or not) the existence of the quantum coherent effects and the quantum coherence induced in the medium, i.e., the qubit transmission line, which are predicted through analytical considerations, consist of integrating Eqs. 3a-3c and 4 of the paper ($n = 1, N$)

$$\dot{R}_x(n) = -\left[\Delta + 8D\sin^2\frac{\alpha_n}{2}\right] R_y(n), \tag{53a}$$

$$\dot{R}_y(n) = \left[\Delta + 8D\sin^2\frac{\alpha_n}{2}\right] R_x(n) - 8\mu\sin^2\frac{\alpha_n}{2} R_z(n), \tag{53b}$$

$$\dot{R}_z(n) = +8\mu\sin^2\frac{\alpha_n}{2} R_y(n), \tag{53c}$$

and

$$\ddot{\alpha}_n + \chi\left[\Omega^2 + \mu R_x(n) + D R_z(n)\right]\sin\alpha_n = \beta^2\delta a_n, \tag{54}$$

where $\delta\alpha_n = \alpha_{n-1} - 2\alpha_n + \alpha_{n+1}$ and the parameters D, Ω, ϵ_p, μ, Δ, and χ are defined in the paper as functions of the interaction matrix elements V_{ij} $(i, j = 1, 2)$, the energy difference between the ground and the first excited

level, $E_1 - E_0$, and the discrete vector potential coupling parameter β. The earlier system of ordinary differential equations is integrated in time with a standard fourth order Runge-Kutta algorithm with constant time-step Δt, typically 10^{-3}. Such small time-steps, and even smaller sometimes, are required to conserve up to high accuracy the total and partial probabilities as the compound system of qubits and the electromagnetic vector potential evolve in time. In all the simulations, periodic boundary conditions are used. Due to the particular shape (Lorentzian) of the EM vector potential pulse and the population inversion pulse in the qubit subsystem, very large systems with $N = 2^{13} = 8192$ and $N = 2^{14} = 16384$ have been simulated to diminish as much as possible the effects from the ends (i.e., the interaction of the pulse tail with itself, as it could be in the case of periodic boundary conditions). In some cases, it is necessary to simulate even larger systems, with $N = 50,000$. In the figures presented in the paper and here only part of these arrays is shown, for clarity. In order to observe two-photon self-induced transparent (TPSIT) pulses $a_n(t)$ and the induced quantum coherent population inversion pulses $R_z(n;t)$, the following initial coditions are implemented: for the former, the analytically obtained solution resulting for the given set of parameters, while for the latter all the qubits are set to their ground, E_0, state. In terms of the Bloch vector variables R_i, $i = x, y, z$, that initial condition is specified as:

$$R_{x,y}(t=0) = 0, \qquad R_z(t=0) = -1, \tag{55}$$

for any $n = 1, ..., N$. In that case, the above mentioned pulses exist for velocities less than the corresponding limiting velocity for TPSIT media, i.e.,

$$v < c = \beta^2 \frac{k}{\omega} = 2\beta^2 \frac{k}{\Delta}. \tag{56}$$

The last equality is valid only when *the two-photon resonance condition* $\omega = \Delta/2$ is satisfied. In Eq. (8), k and ω denote the wavenumber and frequency of the carrier wave of the electromagnetic vector potential; while the latter is determined by the two-photon resonance condition, the former may vary within an interval which is restricted by the qubit-parameter-dependent condition

$$2\chi^2(V_{11} + V_{00}) < (E_1 - E_0)^2, \tag{57}$$

that guarantees the wavenumber k being real.

The role of decoherence in TPSIT in superconducting quantum metamaterials like the one investigated here can be observed in Figures 1, 2, and 3, in which the amount of decoherence, quantified by the factor γ, takes the values 0, 0.01 and 0.1, respectively. The decoherence factor γ depends solely on the interaction matrix elements V_{ij}; it is given by

$$\gamma = 2\frac{V_{11} - V_{00}}{V_{10}}, \tag{58}$$

i.e., it is equal to zero for $V_{00} = V_{11}$ while it assumes non-zero values for $V_{11} > V_{00}$. The Figures 1-3 show snapshots of $R_z(n;t)$ and $a_n(t)$ at several instants from $t = 0$ to $t = 168$, which are separated by 14 time units, along with the corresponding analytical solutions. All these profiles but the first are shifted downwards to avoid overlapping, while only part of the array is shown for clarity. Note that time increases downwards. In Figure 1, for $\gamma = 0$, the $a_n(t)$ pulse is observed to excite a population inversion pulse $R_z(n;t)$ in the qubit subsystem (Figure 1a). The amplitude of $R_z(n;t)$ gradually increases until it attains its maximum value close to unity, while at the same time it propagates to the right with velocity v'. In the figure, that occurs for the first time at $t \simeq 70$ time units; subsequently it evolves in time while it keeps its amplitude almost constant for at least 56 time units. After that, its amplitude starts decreasing until it is completely smeared (not shown). During the time interval in which the amplitude of the $R_z(n;t)$ pulse is close to the predicted one (i.e., close to unity), the metamaterial is considered to be in the *almost quantum coherent* regime. Note that at about $t = 84$ a little bump starts to appear which grows to a little larger (*"probability bump"*) which gets pinned at a particular site at $n \sim 300$. Note that another such bump appears at $n = 0$ due to the initial "shock" of the qubit subsystem because of the sudden onset of the $a_n(t)$ pulse. A comparison of the numerical $R_z(n;t)$ profiles with the analytical ones reveals that the velocity of propagation v', which is the same as that of the numerical $a_n(t)$ pulse (Figure 1b), is slightly larger than the predicted one v ($v' > v$). The corresponding profiles for very weak decoherence, in which the value of the decoherence factor $\gamma = 0.01$, are shown in Figure 2. For that weak decoherence, there are only slight differences in comparison with Figure 1 which actually cannot be observed in the scale of the figures.

In Figure 3, the decoherence factor has acquired a substantial value of $\gamma = 0.1$, so that its effects are now clearly observed in the population inversion pulse $R_z(n;t)$, while the vector potential pulse $a_n(t)$ does not seem to be affected. In Figure 3a, while the $R_z(n;t)$ is still excited by the vector potential pulse $a_n(t)$, it has a very low amplitude compared with that of the analytically predicted form. Indeed, that amount of decoherence only slightly changes the analytical

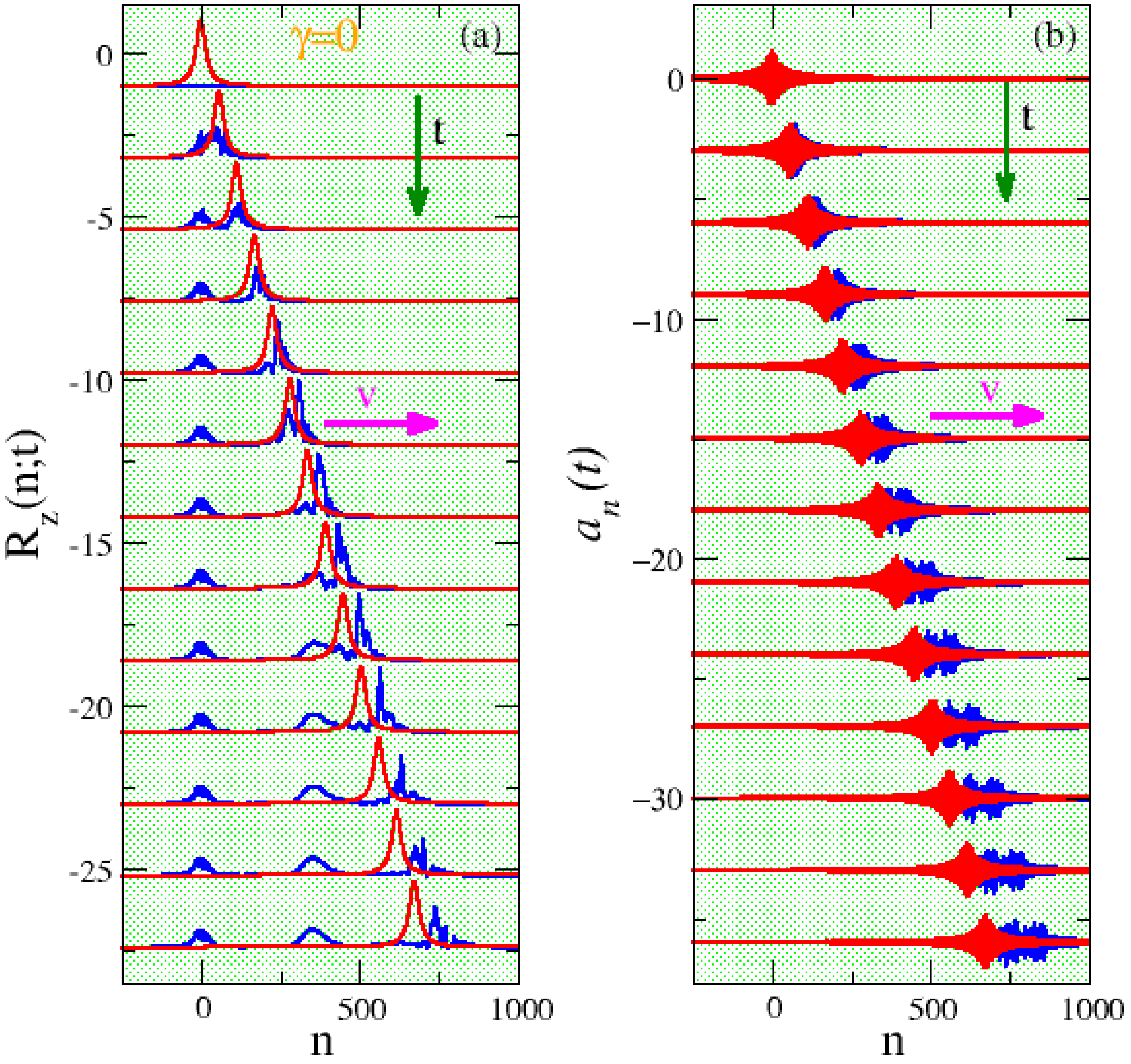

FIG. 1: **Numerical validation of the analytical expressions for two-photon self-induced transparent (TPSIT) propagating pulses in superconducting quantum metamaterials (SCQMMs) without decoherence. a,** Snapshots of the population inversion pulse $R_z(n;t)$, excited by the induced quantum coherence in the qubit subsystem by the electromagnetic vector potential pulse, in the absence of decoherence ($\gamma = 0$); the pulse propagates to the right (time increases downwards). **b,** Snapshots for the corresponding evolution of the electromagnetic vector potential pulse $a_n(t)$, that exhibits significant broadening while its amplitude decreases as time passes by; the numerical and analytical pulses are shown in blue and red color, respectively. Parameter values: $\chi = 1/4.9$, $\beta = 6$, $V_{00} = V_{11} = 1$ ($\gamma = 0$), $V_{01} = V_{10} = 0.7$, $E_1 - E_0 = 3$, and $v/c = 0.7$.

$R_z(n;t)$ pulse and that change is hardly visible in the scale of the figure. Note that the speed of the $R_z(n;t)$ is the same as in the case without decoherence (which is also the same with that of the $a_n(t)$ pulse, in Figures 1-3); even the unwanted "probability bumps" appear at about the same locations with almost the same amplitude and shape independently of the amount of decoherence.

Next, the possibility for the existence of propagating two-photon superradiant pulses in SCQMMs for large values

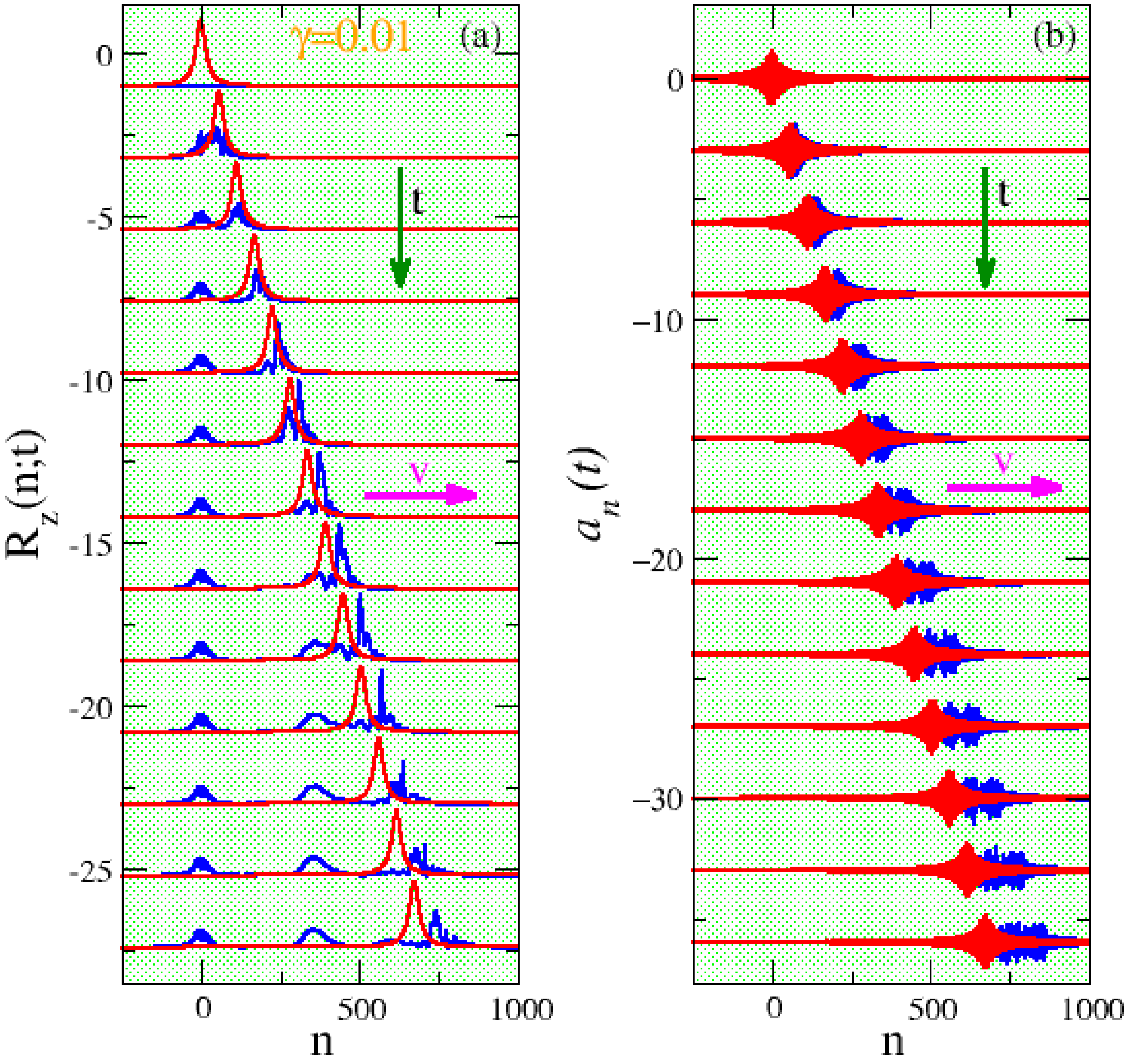

FIG. 2: **Numerical validation of the analytical expressions for two-photon self-induced transparent (TPSIT) propagating pulses in superconducting quantum metamaterials (SCQMMs) in the presence of weak decoherence.** **a,** Snapshots of the population inversion pulse $R_z(n;t)$, excited by the induced quantum coherence in the qubit subsystem by the electromagnetic vector potential pulse, in the absence of decoherence ($\gamma = 0.01$); the pulse propagates to the right (time increases downwards). **b,** Snapshots for the corresponding evolution of the electromagnetic vector potential pulse $a_n(t)$, that exhibits significant broadening while its amplitude decreases as time passes by; the numerical and analytical pulses are shown in blue and red color, respectively. Parameter values: $\chi = 1/4.9$, $\beta = 6$, $V_{00} = 0.998$, $V_{11} = 1.002$, $V_{01} = V_{10} = 0.7$, $E_1 - E_0 = 3$, and $v/c = 0.7$.

of β is numerically explored (Figure 4). In order to obtain that figure, the qubit subsystem is initialized with all the qubits in their excited state, while the vector potential pulse assumes its analytically predicted form for the selected parameter set. A very large array with $N = 50,000$ is simulated, and the initial position of the (center of) $a_n(t)$ pulse is at $n = -18,750$. The subsequent evolution produces the snapshots presented in Figure 4, from $t = 0$ up to $t = 300$

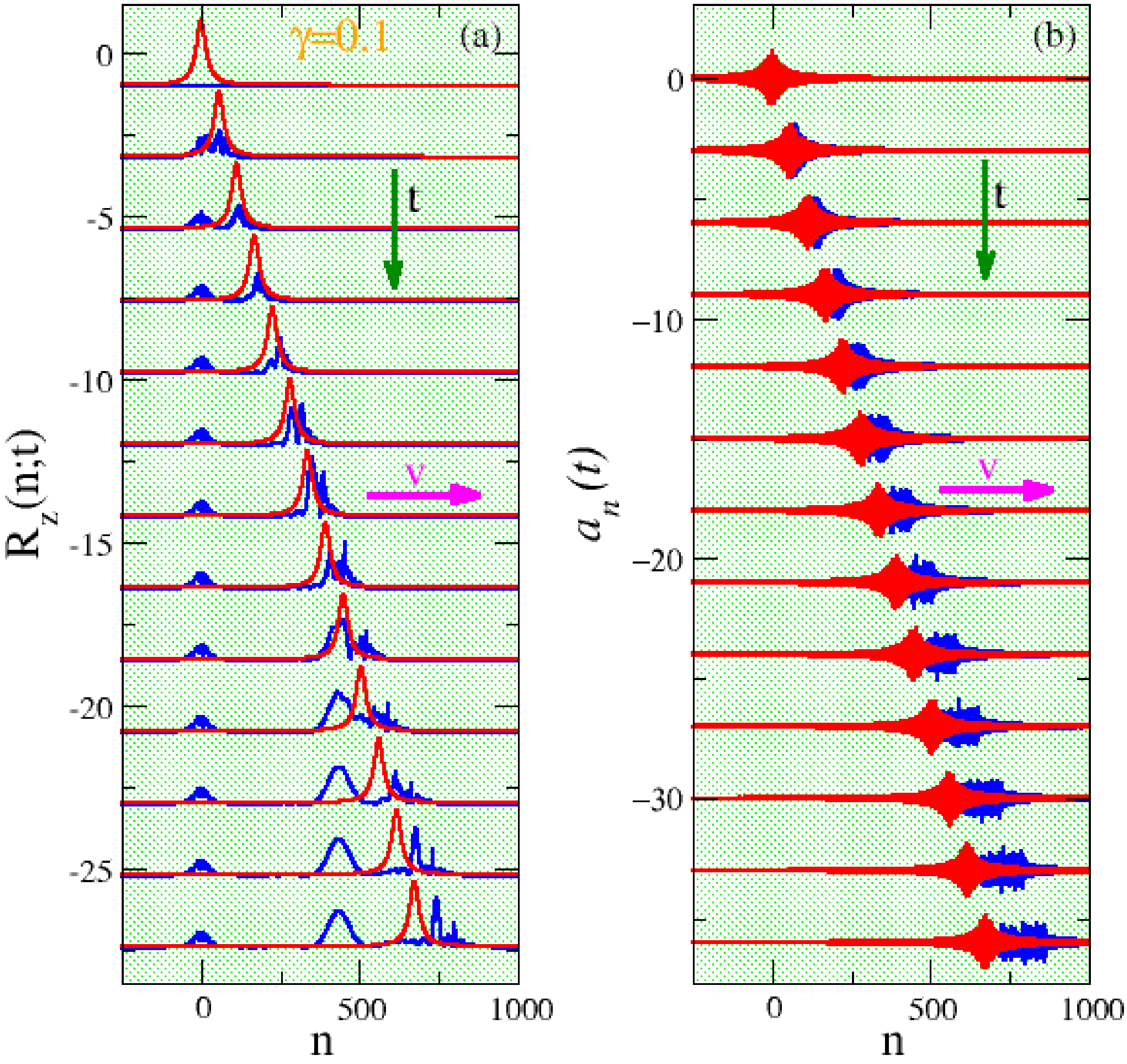

FIG. 3: **Numerical validation of the analytical expressions for two-photon self-induced transparent (TPSIT) propagating pulses in superconducting quantum metamaterials (SCQMMs) in the presence of substantial decoherence. a,** Snapshots of the population inversion pulse $R_z(n;t)$, excited by the induced quantum coherence in the qubit subsystem by the electromagnetic vector potential pulse, in the absence of decoherence ($\gamma = 0.1$); the pulse propagates to the right (time increases downwards). **b,** Snapshots for the corresponding evolution of the electromagnetic vector potential pulse $a_n(t)$, that exhibits significant broadening while its amplitude decreases as time passes by; the numerical and analytical pulses are shown in blue and red color, respectively. Parameter values: $\chi = 1/4.9$, $\beta = 6$, $V_{00} = 0.98$, $V_{11} = 1.02$, $V_{01} = V_{10} = 0.7$, $E_1 - E_0 = 3$, and $v/c = 0.7$.

time units (time increases downwards). These snapshots are separated in time by 20 time units and they are displaced vertically to avoid overlapping. The population inversion $R_z(n;t)$ and the electromagnetic vector potential pulse $a_n(t)$ are shown in Figures 4a and 4b, respectively. In that simulation, the parameter β is much larger than that used in Figure 3b of the paper, i.e., here $\beta = 30$. The other parameters, except the pulse speed, here $v = 2.5 > c$, are the

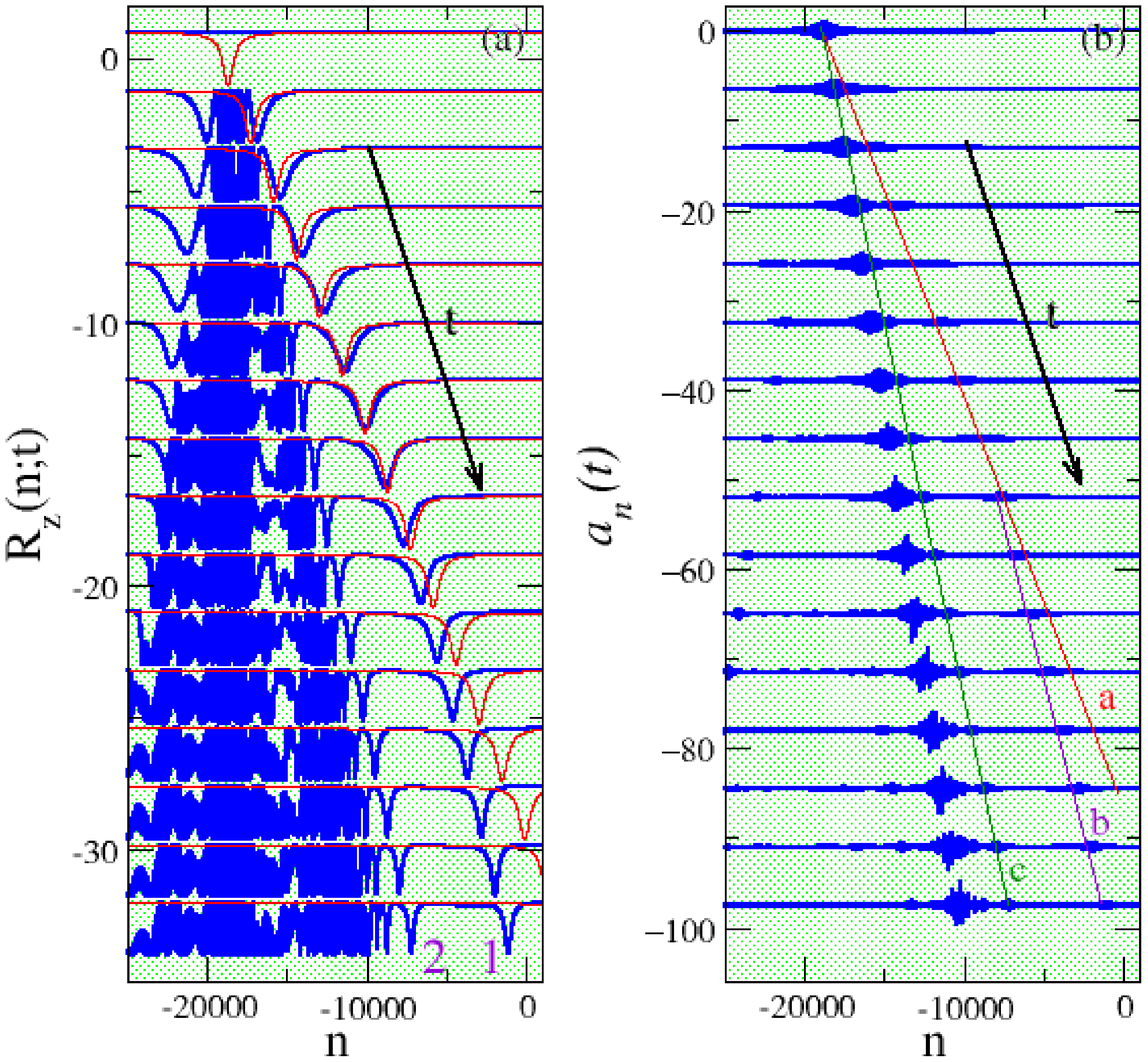

FIG. 4: **Numerical exploration of the propagation of two-photon superradiant (TPSRD) pulses in superconducting quantum metamaterials (SCQMMs) for large values of** β**. a,** Snapshots of the population inversion $R_z(n;t)$ induced in the qubit subsystem by the electromagnetic vector potential pulse, $a_n(t)$ (blue), in the absence of decoherence ($\gamma = 0$). The analytically predicted forms are shown in red color. **b,** Snapshots for the corresponding vector potential pulse $a_n(t)$ at the same time-instants. Parameter values: $\chi = 1/5$, $\beta = 30$, $V_{00} = V_{11} = 1$, $V_{01} = V_{10} = 0.8$, $E_1 - E_0 = 3$, and $v/c = 2.5$

same as those used in Figure 3b of the paper. The spatio-temporal evolution of both pulses reveals a rather complex pattern, that is discussed below. In Figure 4b, the $a_n(t)$ pulse breaks into several pulses of different amplitudes and velocities; the most important are discernible along the green (c) and the purple (b) solid lines. In the same subfigure, the red (a) solid line indicates the analytically predicted pulse speed for two-photon superradiance in superconducting quantum metamaterials. The $a_n(t)$ pulses emerging from the initial one affect the qubit subsystem in a peculiar and complex way: First of all, a very well-defined $R_z(n;t)$ pulse is observed which follows the analytical solution (in red)

for quite some time (about ~ 140 time units). After that time the pulse (whose last snapshot is numbered 1) slows down while it gets narrower while it is propagating together with the $a_n(t)$ pulse along the purple (b) line. Note that around the initial position of the $a_n(t)$ pulse, the population inversion $R_z(n;t)$ exhibits a region of strong variability within its extreme values ± 1 which expands in the course of time. At $t \sim 100$ a second $R_z(n;t)$ pulse is observed to emerge from the strong variability region, which is significantly narrower than the analytical solution. That second pulse ((whose last snapshot is numbered 2) propagates slower than the first one (1), along with the $a_n(t)$ pulse which can be observed along the green line (c) of Figure 4b.
